# Rapid Finger-Pump Microfluidic Paper-Based Assay Platform for Monitoring Calcium Ions in Human Biofluids

**DOI:** 10.3390/bios16040183

**Published:** 2026-03-24

**Authors:** Kuan-Hsun Huang, Chin-Chung Tseng, Chia-Chun Lee, Cheng-Xue Yu, Lung-Ming Fu

**Affiliations:** 1 Department of Engineering Science, National Cheng Kung University, Tainan 701, Taiwan; n96104496@gs.ncku.edu.tw (K.-H.H.); n98121509@gs.ncku.edu.tw (C.-X.Y.); 2 Division of Nephrology, Department of Internal Medicine, National Cheng Kung University Hospital, College of Medicine, National Cheng Kung University, Tainan 704, Taiwan; chinchun@mail.ncku.edu.tw (C.-C.T.); n045740@mail.hosp.ncku.edu.tw (C.-C.L.); 3 Institute of Clinical Medicine, College of Medicine, National Cheng Kung University, Tainan 704, Taiwan

**Keywords:** CKD, microfluidic, blood, serum, urine, calcium ions

## Abstract

Chronic kidney disease (CKD) is a progressively worsening condition that erodes renal function over time, reduces quality of life, and can ultimately culminate in kidney failure with far-reaching systemic complications. In addition to reduced filtration, worsening kidney function disrupts mineral homeostasis and leads to CKD–mineral and bone disorder (CKD-MBD). Dysregulated calcium handling and maladaptive endocrine responses contribute to bone pathology and increase cardiovascular calcification risk; therefore, serial calcium monitoring remains clinically relevant for longitudinal CKD management. Conventional calcium measurements are typically obtained with centralized analyzers or laboratory assays (e.g., colorimetry and electrode/optical readouts). Despite high accuracy, the required instrumentation, controlled operating conditions, and pretreatment steps complicate rapid point-of-care deployment, especially when only microliter-scale biofluids are available. Accordingly, this study develops a finger-actuated microfluidic colorimetric platform capable of determining calcium ion concentrations in human biofluids, such as whole blood, serum, and urine. The platform integrates a three-dimensional PMMA/paper microchip with a compact reader that maintains stable temperature control while enabling CMOS-based optical detection. With just 6 μL of sample, a brief finger press propels the biofluid across an internal filtration layer, generating serum or cleaned urine that subsequently reacts with a pre-deposited murexide reagent. Under optimized conditions (1.6% reagent, 50 °C, 3 min), the signal follows a strong logarithmic relationship with calcium concentration (Y = 47.273 ln X + 28.890; R^2^ = 0.9905), supporting quantification over 1–40 mg/dL and a detection limit of 0.2 mg/dL. Across 80 clinical CKD specimens spanning serum, whole blood, and urine, results aligned closely with the NM-BAPTA reference assay, with R^2^ values exceeding 0.97.

## 1. Introduction

The global incidence of chronic kidney disease (CKD) is increasing, imposing a significant burden on healthcare systems worldwide. CKD significantly impairs the quality of life of patients and is associated with many adverse health complications, including kidney failure, cardiovascular disease, anemia, and bone metabolism disorders [1]. CKD is recognized through a combination of laboratory testing (blood and urine), medical imaging, and, when necessary, kidney biopsy. Beyond these diagnostic measures, physicians must also follow a cluster of common comorbidities—diabetes, gout, hypertension, and nutritional deterioration—since these secondary conditions often complicate the disease course and influence clinical decisions.

CKD is generally classified into five stages, with stage 1 indicating mild kidney damage and stage 5 indicating end-stage renal failure. Early CKD is frequently clinically silent, and initial manifestations may be subtle or nonspecific. With disease progression, nonspecific symptoms may emerge, including sleep disturbance, anorexia, nausea, emesis, and persistent flank or lower-back discomfort [2]. Once the disease reaches its later stages, more complex complications usually emerge, including hypertension, glomerulonephritis, nephrolithiasis, and diabetes. Thus, early prevention and timely medical action can meaningfully slow CKD progression, and by doing so, lessen the substantial health burden the disease ultimately imposes [3,4].

Calcium is distributed throughout many tissues in the human body, and shifts in its availability can influence a wide range of physiological processes. In healthy individuals, serum calcium is usually maintained within a narrow physiological band (8.8–10.4 mg/dL) [5]. When concentrations drift downward, clinicians often observe a higher frequency of CKD in practice and across reported population studies [6]. Accordingly, individuals at risk for CKD are often advised to manage dietary mineral intake and undergo periodic monitoring of circulating calcium (Ca^2+^) as part of longitudinal care. Several analytical techniques have been developed for Ca^2+^ detection, including mass spectrometry [7], colorimetric assays [8], potentiometric sensors [9], fluorescence techniques [10,11], electrochemical detection [12], ion-selective electrodes [13], and optical detection [14]. While these laboratory approaches can deliver reliable data, they often depend on specialized equipment and demanding manual steps, making them impractical for use at the point-of-care (PoC) testing.

Recently, microfluidic devices have received increasing attention in a wide range of fields because of their excellent biocompatibility and high level of functional integration. The literature contains many studies on microfluidic chips, lab-on-a-chip (LoC) systems, and microfluidic paper-based devices (μPADs) [15,16,17,18,19,20,21,22] for applications, ranging from biological research [23,24,25] to clinical diagnostics [26,27,28], environmental monitoring [29,30,31], chemical analysis [32,33,34], forensic science, and food safety testing [35,36]. Microfluidic assays continue to draw attention because they enable rapid measurements while consuming only minute sample volumes [37,38,39,40]. Recently, these characteristics have fueled growing interest in developing microfluidic-based calcium quantification systems for human biofluids [41,42,43,44,45,46,47]. Therefore, rapid and reliable assessment is clinically valuable for PoC testing applications and long-term CKD management.

Microfluidic platforms that integrate digital imaging, potentiometric sensing, or fluorescence detection provide reliable and low-cost strategies for measuring calcium ion concentrations in biological samples [41,42,43,44,45,46,47]. Huang et al. [41] reported a multiplexed microfluidic platform that combined fluorescence and ELISA modules, enabling parallel readout of extracellular Ca^2+^ and several hormones. Their system provided fine temporal resolution, covering 0.2–2 mM Ca^2+^ with an approximate detection limit of 0.2 mM. In a separate effort, Lewińska and co-workers [45] introduced a dual-channel μPAD that quantified Ca^2+^ and Mg^2+^ directly from human serum by employing o-cresolphthalein complexone and xylidyl blue dyes as the colorimetric indicators. The device showed a comparable performance to clinical reference assays, with a detection limit of 0.09 mM for Ca^2+^ and a detection range of 0.1–2.6 mM. Li et al. [47] designed a flexible potentiometric detector with integrated Bluetooth communication for sweat electrolyte sensing. The platform exhibited a strong quantitative agreement with ion chromatography for Ca^2+^ detection in real-time, on-body applications, with an effective detection limit of approximately 0.05 mM and an operating range covering physiological concentrations.

Despite significant technological advancements in recent microfluidics research, many fluorescence- and ELISA-based schemes still rely on bulky optical components and external readout modules, limiting their practicality outside of central laboratories. These constraints make it difficult to use such systems in PoC situations where testing is expected to occur outside a laboratory, such as routine follow-up at smaller clinics or at home. This situation illustrates the importance of compact and inexpensive platforms that can quantify calcium accurately without auxiliary instrumentation.

This study establishes a finger-actuated microfluidic paper-based assay capable of quantifying Ca^2+^ directly from human biofluids. A three-dimensional PMMA–paper layout guides filtration, reaction development, and optical capture within a compact structure that can be driven by a light press of the fingertip. Once the chromogenic region reaches the optimal conditions for chelation, only a few microliters of sample are required, and meaningful results emerge within minutes. Across the physiologically relevant concentration span, the calibration response remains exceptionally stable. More importantly, the performance persists when tested against genuine clinical variation: measurements obtained from 80 CKD specimens—including whole blood, serum, and urine—show a strong agreement with the NM-BAPTA reference method (R^2^ > 0.97). These features support practical deployment in decentralized settings where timely electrolyte readouts carry direct clinical consequence.

## 2. Materials and Methods

### 2.1. Sample and Reagent Preparation

A 100 mg/dL calcium stock was obtained by dissolving 0.5893 g Ca(NO_3_)_2_·4H_2_O (Sigma-Aldrich, St. Louis, MO, USA) in 100 mL deionized (DI) water. Serial dilutions of this stock with DI water were then used to prepare working control solutions at 1, 1.5, 2, 2.5, 5, 10, 20, 30, and 40 mg/dL. At National Cheng Kung University Hospital (NCKUH), 8.6–10.0 mg/dL defined the normal serum calcium interval, while values <6.5 mg/dL or >13.0 mg/dL indicated clinically concerning deviation. Because clinically reportable concentrations typically fall within 0.8–20 mg/dL, the analytical window was set to 1–40 mg/dL to capture both physiological levels and pathophysiological extremes.

**Scheme 1:**
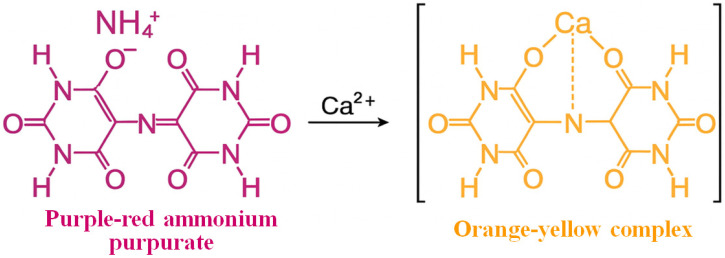
Colorimetric reaction of ammonium purpurate with Ca^2+^.

Ammonium purpurate (murexide; NH_4_C_8_H_4_N_5_O_6_, Sigma-Aldrich, St. Louis, MO, USA.) was used as the chromogenic reagent and prepared as an alkaline aqueous solution for Ca^2+^ complexation. It is widely used as an indicator because it undergoes distinct color changes in aqueous solutions depending on the pH of the environment, including yellow under acidic conditions, reddish-purple in weakly acidic media, and bluish-purple in alkaline environments. For selective sensing, Ca^2+^ specificity relies on maintaining the medium in a strongly alkaline region (around pH 11.3) [48]. At high pH, murexide forms a Ca^2+^–ligand complex accompanied by a distinct color transition, enabling colorimetric quantification under alkaline conditions. In practice, the color deepens as Ca^2+^ increases, as illustrated in Scheme 1.

The amount of reagent deposited on paper was adjusted by screening three different concentrations of ammonium purpurate. Ammonium purpurate was formulated at three loadings—0.16, 0.18, and 0.20 g—each dissolved in 8 mL of DI water. Dissolution was promoted at 75 °C with stirring for 20 min; after cooling to 25 °C, the pH was adjusted to 11.3 by adding 100 μL of 1 M NaOH (Sigma-Aldrich, St. Louis, MO, USA.). An alkaline solution is drop-cast onto the paper detection zone and air-dried to form an alkali-impregnated reagent pad. The reagent-loaded microchip is stored under a low-temperature vacuum to safeguard activity. Contact with the sample restores mobility to the entrapped alkali, sharply elevating pH at the reaction site. This spatially confined alkalinity sustains Ca^2+^ chelation and produces a rapid, consistent signal.

### 2.2. 3D Microfluidic Finger Pump Device

**Figure 1:**
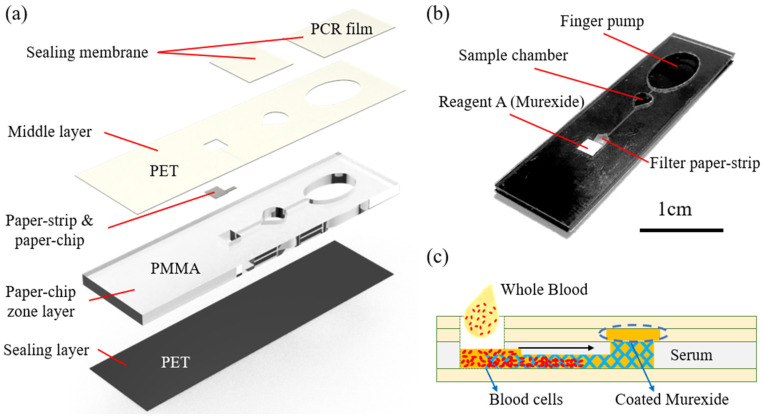
Shows (**a**) the functional components of the finger pump microchip and (**b**) a photograph of the completed microchip. (**c**) The microchip’s configuration and operation are depicted in a cross-sectional view.

Figure 1 summarizes the device architecture, including the exploded layout, assembled microchip, and a cross-sectional view prior to encapsulation. The polymethyl methacrylate (PMMA) microchip (42 mm × 11 mm × 2 mm, purchased from a local stationery store) is organized into four working regions—finger pressure pump, filtration zone, microchannel, and detection zone—arranged to guide sample handling from entry to readout. Unlike conventional paper-based devices that rely solely on spontaneous wicking, this layout is designed to accommodate complex biofluids such as whole blood and patient urine. In these matrices, capillary transport can be slowed or become unstable, and suspended solids can partially clog the pore network before a stable filtrate is formed. Therefore, a finger pump can provide brief, metered pressure pulses to trigger the filtrate into the filter paper and rapidly produce a clarified solution for subsequent colorimetric reactions.

For biofluid assays, the sample is deposited in the filtration zone and sealed with a PCR film (MSB1001, Genmall Biotech. Co., Ltd., Tainan, Taiwan) that serves as the finger-pump diaphragm. A light press drives the fluid into the filter-paper strip beneath; once the paper is wetted, lateral wicking takes over and capillary forces carry the sample smoothly into the detection region. This pressure-assisted filtration is central to the platform’s unique contribution: it enables on-chip purification from microliter-scale whole blood and heterogeneous urine without centrifugation, pumping hardware, or off-chip pretreatment, thereby widening the practical scope of paper-based colorimetry to clinically relevant biofluids. As the sample wicks forward, the porous paper functions as a graded filter, holding back larger particulates and intact cells so that a clarified fraction of serum or urine reaches the detection zone. That zone is preloaded with ammonium purpurate, so color development begins immediately when the filtrate arrives. Because the reagent pad is pre-alkalinized after drying, neither NaOH addition nor on-site pH adjustment is required. A single sample droplet rehydrates the on-chip reagents and triggers complexation. The reacted chip is then read in a portable device (Self-developed) to capture the Ca^2+^ signal for quantification.

Each calibration point was measured in five independent replicates (*n* = 5) and is reported as mean ± standard deviation (SD). For clinical samples, five measurements were obtained per sample using separate wafers; the resulting mean value was then compared with the reference assay. Linear least-squares regression was used to determine the correlation coefficient (R^2^).

### 2.3. Hand-Held Detection System

**Figure 2:**
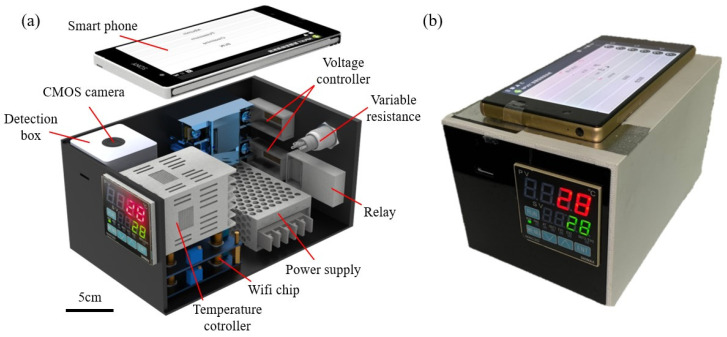
(**a**) The functional components of the detection system. (**b**) Photograph of the calcium ion detection system.

Figure 2 shows the handheld device that was used to identify Ca^2+^. The device measured 193 mm × 128 mm × 128 mm and was powered by a 12 V/5 V supply (RID-85A, Dunhua Electronics Co., Taichung, Taiwan). The detection module was built around a CMOS imaging unit with a dedicated lens (HYNIX HI843, Guangzhou Xincheng Information Technology Co., Guangzhou, China), which captured high-resolution images from the reaction zone of the microchip; an LED light source (Opto Plus LED Corp., New Taipei, Taiwan) for producing uniform illumination; and a heating plate, regulated by a temperature controller (PXR-4, Fuji Electric Co., Taipei, Taiwan) to maintain stable reaction conditions during the measurement process. To ensure stable operation, the circuit employs relay switches, a buck converter, and a voltage/temperature combination control switch. One miniature display panel (PLC1601BW, Palm-Tech Co., Kaohsiung, Taiwan) was used for readout, and a cooling fan (DF8015UB, Jieyi Technology, New Taipei, Taiwan) together with a manual voltage adjustment knob allowed fine tuning of thermal and electrical conditions during use. In the detection process, images captured by the CMOS module were transmitted wirelessly to a smartphone. A custom application processed the pixel color distribution and converted it into a quantitative Ca^2+^ value based on an established calibration curve. For each measurement, mean R, G, and B intensities were obtained from the predefined region of interest, and the composite metric (R + G − B) was computed for calibration.

## 3. Results

### 3.1. Effects of Reaction Time and Reaction Temperature

**Figure 3:**
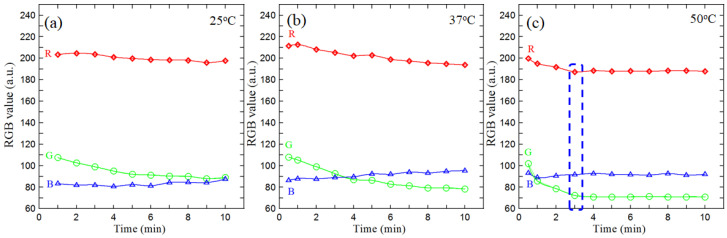
Reaction times for Ca^2+^ detection at different temperatures: (**a**) 25 °C, (**b**) 37 °C, and (**c**) 50 °C.

For the proposed platform, the reagent–sample reaction time was a primary determinant of color-complex stability and, consequently, the accuracy and precision of quantitative readout. Figure 3a shows the time-varying intensity signals of the color complex R(ed), G(reen), and B(lue) at 25 °C using 1.5% reagent and 20 mg/dL Ca^2+^. At 25 °C, the RGB channels also show very subtle changes over time. Each channel exhibits minimal drift, whereas the overall color evolution is slow and does not converge to a stable endpoint within the observation window. Such kinetics are consistent with slower chelation and restricted ion transport in the paper network under room-temperature conditions. Accordingly, room-temperature operation (25 °C) did not yield a stable endpoint within the tested window and was not adopted for quantitative measurements.

As shown in Figure 3b,c, the reaction rates increased markedly with an increasing temperature. In accordance with the Arrhenius equation [49], increasing the temperature increased the fraction of molecules with sufficient energy to overcome the activation barrier, thereby accelerating the interaction between the Ca^2+^ ions and the ligand (murexide). Furthermore, the viscosity of the aqueous control samples decreased as the temperature increased, which enhanced the ionic diffusion and capillary flow, resulting in a more uniform reagent distribution. At 50 °C (Figure 3c), the color intensity stabilized within ~3 min, indicating that the chromogenic reaction was effectively complete over this interval. This temperature–time combination also avoided thermal stress on the paper substrate and maintained reagent integrity, so it was adopted in the later experiments as the standard operating condition.

**Figure 4:**
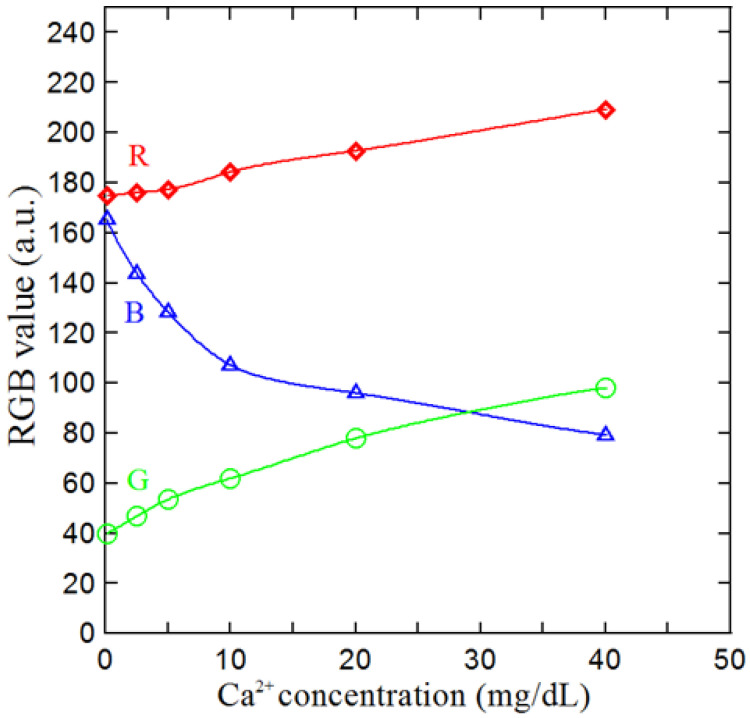
Concentration dependence of the R, G, and B channel intensities and the composite metric (R + G − B) for Ca^2+^ under the selected reaction conditions (50 °C, 3 min).

Figure 4 shows the R, G, and B signals recorded under the selected reaction conditions of 50 °C, using 1.5% reagent for 3 min, with Ca^2+^ concentrations ranging from 1 to 40 mg/dL. The R and G signals rose progressively with concentration, while the B signal declined. These trends reflect a red shift in the absorption profile of the dye–metal complex: the dominant peak moves from the blue region (~450 nm) toward longer visible wavelengths, consistent with the observed transition in color from light blue to reddish-brown. Such spectral movement suggests changes in the coordination environment (for instance, monodentate toward a more multidentate chelation mode), and the magnitude of this shift relates to both reaction progression and metal ion abundance. Because the R, G, and B channels each responded systematically to concentration, the composite R + G − B value was selected as the signal metric for calibration and quantitative interpretation in the subsequent experiments. A composite metric was used instead of a single channel. Because the murexide–Ca^2+^ reaction increases R and G while decreasing B, (R + G − B) amplifies concordant trends and subtracts the opposing contribution, improving readout stability. As a result, the composite readout typically yields a larger net response than any individual channel and preserves a clean monotonic dependence on concentration. In addition, combining channels reduces the likelihood that quantification will be dominated by channel-specific artifacts (e.g., localized paper texture, uneven reagent distribution, or camera-channel variability), thereby improving the stability of the calibration used for smartphone readout.

### 3.2. Effect of Reagent Concentration and Construction of Calibration Curve

When the reagent concentration was too low, the Ca^2+^ ions did not fully chelate with murexide, and the color development remained weak. Under these conditions, the signal response was noticeably less sensitive. Conversely, an excessive concentration caused the complex color to become saturated and the effects of background interference to increase, which collectively reduced the linearity of the calibration response. To determine the optimal reagent concentration, detection trials were conducted under ideal reaction conditions using murexide concentrations of 1.2%, 1.6%, and 2.0%, along with Ca^2+^ concentrations ranging from 1 to 40 mg/dL.

**Figure 5:**
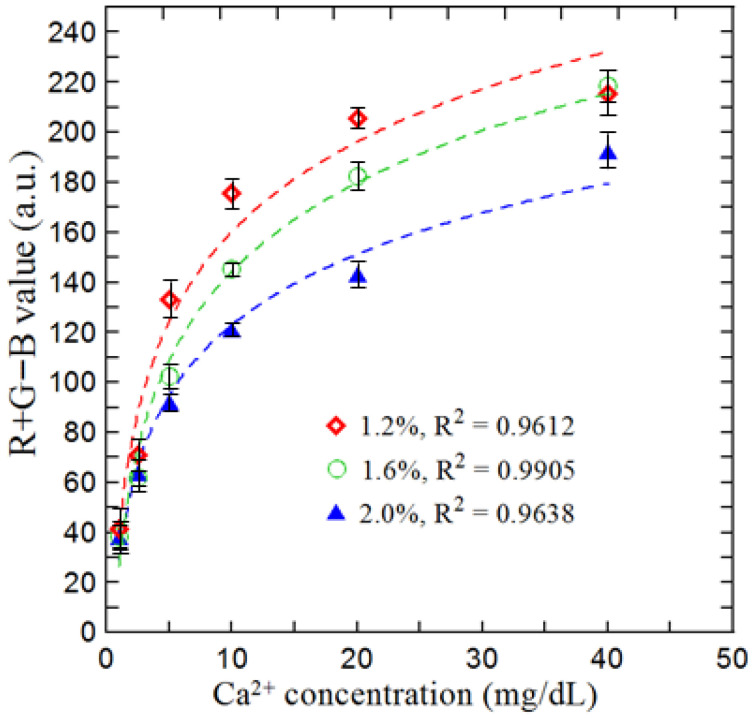
Relationship between R + G − B values and Ca^2+^ concentration (1–40 mg/dL) given Murexide reagent concentrations of 1.2%, 1.6%, and 2.0%.

As shown in Figure 5, the intensity of the colorimetric signal increased significantly with an increasing Ca^2+^ concentration for all three reagent concentrations. Reagent concentration exerted a pronounced influence on assay performance. When the reagent concentration was lowered to 1.2%, the murexide–Ca^2+^ coordination did not proceed completely, and the sensitivity decreased (R^2^ = 0.9612). Among the tested concentrations, 1.6% murexide yielded a markedly more usable behavior. The chromogenic output retained a broad and practical working range, yet the concentration–signal relationship remained tightly linear (R^2^ = 0.9905). Therefore, this condition emerged as the most appropriate choice for routine operation. This condition provided a stable response with a low background and was therefore selected as the operating murexide concentration.

**Figure 6:**
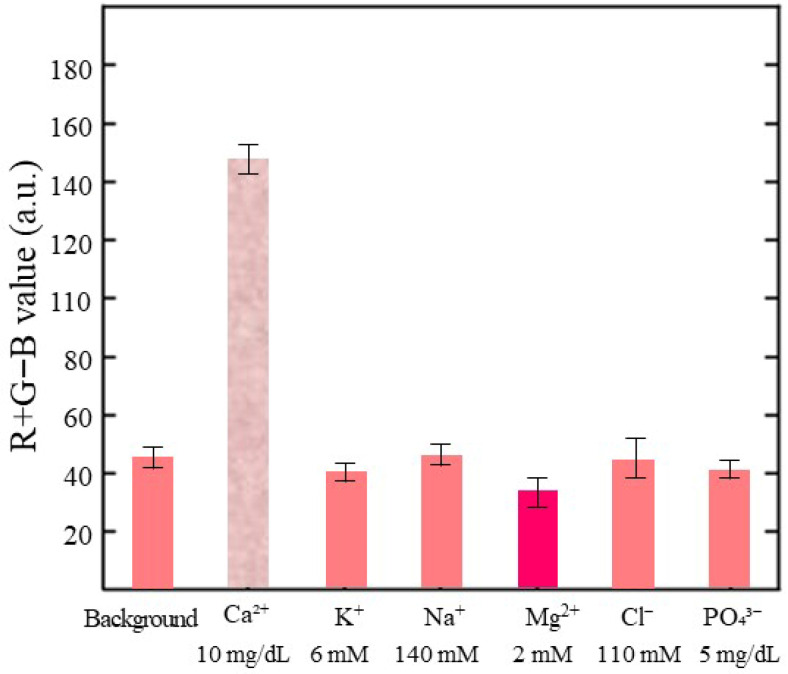
R + G − B values of different electrolyte ions after reaction with murexide reagent.

Human serum presents a high ionic background, dominated by K^+^ (3.5–5.5 mM), Na^+^ (135–145 mM), Mg^2+^ (0.78–1.11 mM), and Cl^−^ (98–108 mM), with inorganic phosphorus typically reported at 2.5–4.5 mg · dL^−1^. Against this backdrop, Ca^2+^ quantification in whole blood must be checked for susceptibility to coexisting electrolytes—K^+^, Na^+^, Mg^2+^, Cl^−^, and phosphate (reported as phosphorus). Platform specificity was evaluated using DI water as the blank, followed by single-ion inputs at physiologically relevant levels: Ca^2+^ (10 mg dL^−1^), K^+^ (6 mM), Na^+^ (140 mM), Mg^2+^ (2 mM), Cl^−^ (110 mM), and inorganic phosphorus (5 mg dL^−1^). As shown in Figure 6, only Ca^2+^ at 10 mg · dL^−1^ produced a conspicuous color response, whereas the other electrolytes remained essentially unchanged. Consistent with this visual contrast, the Ca^2+^ condition raised the R + G − B value by ~222% above the background. By comparison, all non-calcium ions remained near baseline, with R + G − B deviations confined within ±5%, except for Mg^2+^, which decreased the metric by ~12% relative to the background. Importantly, prior work indicates that BAPTA-based sensing membranes retain Ca^2+^ accuracy even in the presence of Mg^2+^ interference [50]. Altogether, the data indicate that the murexide-based readout remains strongly Ca^2+^-selective under physiologically relevant ionic backgrounds.

**Figure 7:**
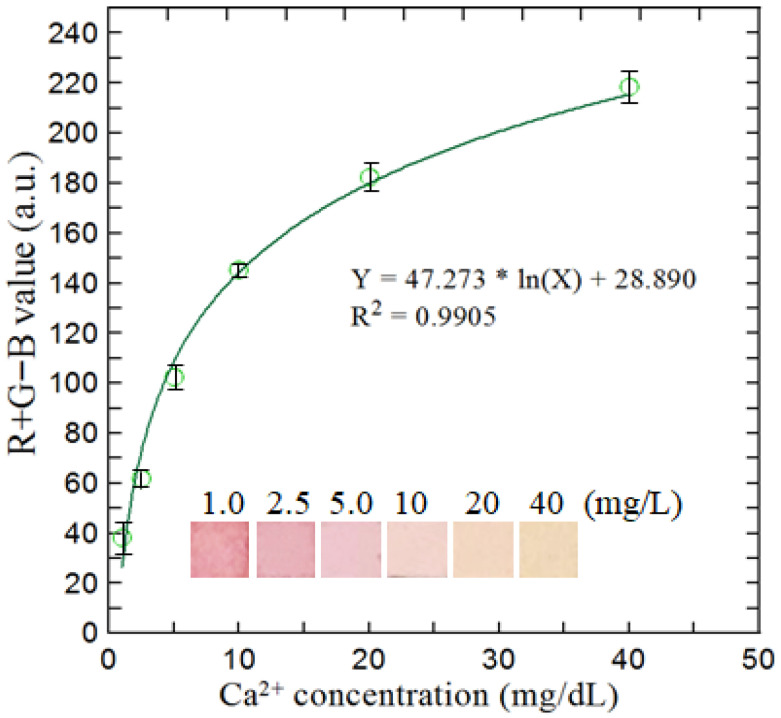
Calibration curve showing variation in R + G − B signal intensity with Ca^2+^ concentration over range of 1–40 mg/dL under optimal reaction conditions.

Control samples with known Ca^2+^ concentrations of 1–40 mg/dL were analyzed using the proposed microfluidic platform under the optimal reaction conditions. Each measurement was repeated five times to ensure the accuracy and reproducibility, and the mean R + G − B value was computed to obtain a representative value. As shown in Figure 7, the R + G − B value (Y) varied with the Ca^2+^ concentration (X) according to the following equation: Y = 47.273 ln(X) + 28.890. The high correlation coefficient (R^2^ = 0.9905) confirmed the suitability of the calibration curve for subsequent quantitative analyses. The logarithmic increase in the R + G − B value with the Ca^2+^ concentration arises from the chelation between the Ca^2+^ ions and murexide reagent, which causes a red shift in the visible spectrum and prompts a color change of the reactant from purplish-red to orange. The analytical detection limit was determined to be 0.2 mg/dL, thereby confirming the excellent sensitivity and reproducibility of the portable detection system for the quantitative determination of low Ca^2+^ levels in clinical samples.

### 3.3. Ca^2+^ Determination in Real-World CKD Patient Samples

To examine how the platform performs under real clinical conditions, we tested 80 specimens collected from CKD patients at NCKUH in Taiwan. The set included 20 random urine samples, 30 whole-blood samples, and 30 serum samples. All materials were obtained under an approved Institutional Review Board protocol (NCKUH IRB #IRB-A-ER-108-527), and written informed consent was obtained prior to enrollment. Each sample was analyzed using the calibration curve established in Figure 7, and the resulting Ca^2+^ values were benchmarked against clinical reference methods. The measured Ca^2+^ concentrations were then compared against values obtained from a routine benchtop analyzer (HITACHI 7180 automated biochemical analyzer, Tokyo, Japan) to evaluate how closely the platform aligns with standard clinical practice.

**Figure 8:**
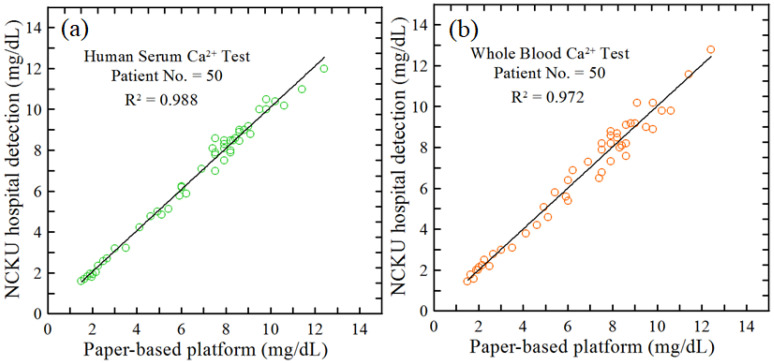
Comparison of results for detecting Ca^2+^ concentration obtained using the proposed microfluidic detection platform and the conventional NM-BAPTA colorimetric method: (**a**) human serum samples and (**b**) whole blood samples.

Validation commenced with the quantification of Ca^2+^ levels in both serum and whole blood. For comparison, each sample was also analyzed using a conventional NM-BAPTA colorimetric assay. Figure 8a,b show a summary of the results. For the serum samples, the detection results showed an excellent agreement (R^2^ = 0.988) with the benchtop measurements over the Ca^2+^ concentration range of 7.5 to 14 mg/dL. To further probe the low-concentration regime, 50 serum datasets were generated by combining the original 30 specimens with two- and threefold dilutions of ten selected samples. Whole blood was processed in the same manner, resulting in 50 samples. Despite the greater variation introduced by dilution and matrix complexity, the values from the chip remained consistent with the reference method (R^2^ = 0.972, as shown in Figure 8b), confirming that the device can still read Ca^2+^ reliably under more demanding sample conditions.

**Figure 9:**
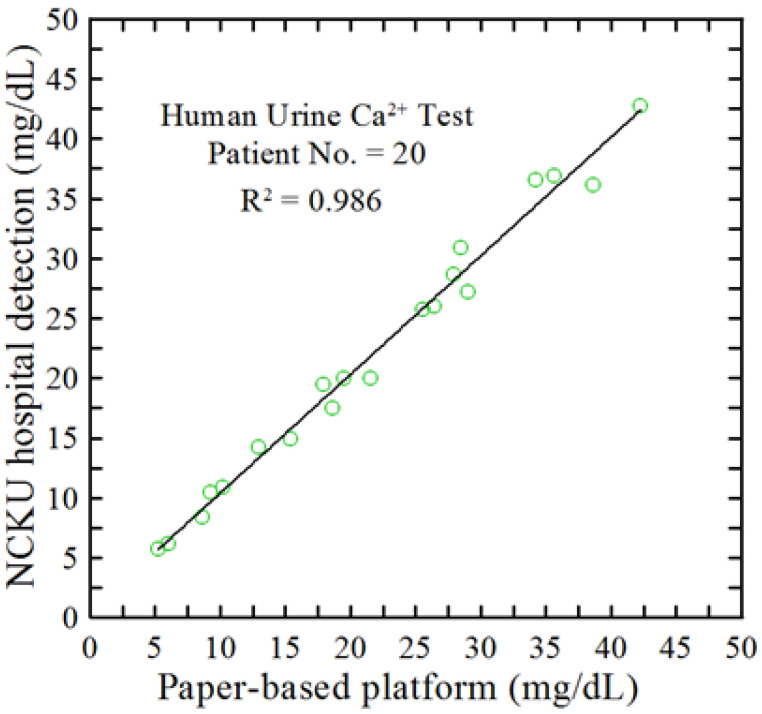
Comparison of Ca^2+^ concentration measurements obtained using the current microfluidic detection platform and the conventional NCKUH method for urinary samples.

Urine specimens were diluted 1:2 (urine: DI water) prior to analysis; aside from this step, real-sample testing required neither pH adjustment nor off-chip pretreatment. The same microchip was used throughout, and its integrated filtration channel cleared turbidity and particulates before the filtrate contacted the murexide reagent. For benchmarking, Ca^2+^ concentrations were also measured using a Beckman Coulter AU5800 (Beckman Coulter, Brea, CA, USA). As shown in Figure 9, each point represents the mean of five replicate measurements collected under identical conditions. Despite pronounced patient-to-patient variability in urine chemistry, the microfluidic readouts closely matched the clinical analyzer (R^2^ = 0.982).

## 4. Discussion

Overall, Figure 8 and Figure 9 indicate strong agreement between the platform and routine clinical measurements across whole blood, serum, and urine within the tested concentration ranges. To contextualize the platform relative to reported Ca^2+^ assays, Table 1 prioritizes point-of-care considerations—workflow burden, infrastructure requirements, and sample handling—alongside analytical performance. Centralized clinical colorimetry and laboratory analyzers still excel in standardized quality control and high-throughput testing. In practice, these advantages often rely on dedicated analyzers, controlled operating conditions, and serum-preparation workflows that may be challenging to replicate in decentralized or resource-limited settings. Fluorescence- or ELISA-enabled microfluidic approaches can offer multiplexing and sensitive readouts but commonly depend on optical components, labeled reagents, or more elaborate workflows. Electrochemical and potentiometric formats are well suited to continuous readout in readily sampled fluids such as saliva or sweat. Nevertheless, routine deployment can be limited by electrode conditioning, drift management, and matrix-specific calibration; in addition, direct whole-blood operation often requires extra measures to mitigate fouling and hematocrit-related effects. By contrast, the present platform couples pressure-assisted on-chip filtration with paper-driven transport. Microliter volumes of whole blood (or clarified urine) are converted into a clear, serum-like filtrate without centrifugation or off-chip pretreatment. That filtrate immediately rehydrates a pre-deposited murexide pad, and the color endpoint is quantified within minutes using a temperature-regulated reader. The primary trade-off is the requirement for a compact heater/imager to standardize endpoint formation, while the readout is inherently endpoint-based rather than continuous.

Table 1 offers a qualitative comparison between the present microfluidic colorimetric assay and representative Ca^2+^ methods reported for human biofluids. The platform is designed for direct Ca^2+^ readout from serum, whole blood, and urine, delivering rapid measurements with little reliance on external instrumentation. Such integration, together with stable analytical performance, supports routine clinical workflows and also fits PoC settings where portability and quick turnaround are decisive. A detailed comparison highlights a key strength of the platform—pressure-assisted on-chip filtration that enables direct handling of clinically relevant biofluids—while also revealing a primary limitation: the need for a compact temperature controller to achieve rapid readout.

## 5. Conclusions

A finger-pump-driven microfluidic paper-based colorimetric platform enables rapid Ca^2+^ detection, targeting a clinically relevant marker in CKD. This microfluidic assay platform combined a three-dimensional PMMA and paper-based microchip with a compact handheld analytical device equipped with precise temperature control and digital imaging capabilities. Under optimized reaction conditions (1.6% murexide, 50 °C, 3 min), the assay produced a clear linear calibration (R^2^ = 0.9905) with a detection limit of 0.2 mg/dL. Experimental trials showed that the system was applicable for the rapid and quantitative measurement of Ca^2+^ concentrations in various human biofluids, including urine, serum, and whole blood. Moreover, the results obtained by the proposed system were in excellent agreement (R^2^ > 0.97) with the measurements obtained using a conventional assay (NM-BAPTA) and a commercial clinical chemistry analyzer.

In the present microchip, a finger-driven pump is paired with on-chip filtration to simplify sample handling and eliminate common pretreatment steps that otherwise depend on powered actuation or centrifugation. Small differences in applied pressure affect how reactions start but have minimal impact on the endpoint signal. Modest temperature drift likewise leaves the final response largely intact. Temperature regulation serves to align reaction timing and measurement consistency, not to change the extent of complex formation. As a result, the workflow remains both rapid and streamlined, requiring only 6 μL of sample. Image capture and RGB-based quantification are performed in real time on a smartphone, easing operator workload and eliminating the need for dedicated analytical hardware. Packaged in a compact, field-deployable format, the assay supports decentralized testing and PoC use.

Overall, the platform provides a low-cost, robust route to Ca^2+^ quantification, suitable for both clinical testing and field deployment. Because the reagent zones and channel structure can be reconfigured when needed, the same framework can be adapted to other biomarker chemistries that share similar reaction principles. In this sense, the present work moves the device architecture closer to a microfluidic format that could support real clinical decision-making at the PoC and continuous physiological monitoring for patients with CKD or related metabolic disorders.

## Figures and Tables

**Table 1:** Qualitative comparison of Ca^2+^ assays, highlighting PoC practicality and analytical performance in the literature.

Author and Year	Sample Type	Real Sample	Detection Reagent, Method & Time	Detection Range & LOD	Strengths/Limitations
Current work	Whole blood, serum, urine	B (30) S (30) U (20)	Murexide Colorimetric 3 min	1–40 mg/dL LOD: 0.2 mg/dL	On-chip filtration enables whole blood/urine; 6 μL; rapid heated endpoint readout. Requires compact heater/imager; endpoint (not continuous) measurement.
NCKU Hospital	Serum, urine	Serum Urine	Colorimetric	S: 0.8–20 mg/dL U: 3.4–285 mg/dL	Clinical-grade standardization and throughput. Requires benchtop analyzer and laboratory workflow; not field/decentralized.
Huang et al. 2020 [41]	Serum	-	Ca^2+^ indicator Fluorescence 3 min	0.2–2 mM LOD: 0.2 mM	Sensitive optical readout; potential multiplexing. Optical components and assay complexity limit low-resource deployment.
Tarara et al. 2023 [42]	Saliva	5	MTB Colorimetric 10 min	3.07–8.42 mg/dL LOD: 0.29 mg/dL	Simple colorimetric workflow; saliva-friendly sampling. Longer assay time; matrix-specific applicability (not whole blood).
Biswas et al. 2023 [43]	Serum	50	Arsenazo III Colorimetric 3 min	8.8–10.4 mg/dL LOD: 4.2 mg/dL	Smartphone-friendly readout. Narrower clinical window and performance constraints; serum-focused workflow.
Aguiar et al. 2023 [44]	Saliva	10	CPC Colorimetric 10 min	1.1–18 mg/dL LOD: 0.32 mg/dL	Low-cost paper microfluidics for noninvasive sampling. Saliva-specific; assay time longer than the proposed platform.
Lewińska et al. 2024 [45]	Serum	10	OCP Colorimetric 10 min	0–40 mg/dL LOD: 0.36 mg/dL	Paper-based multiplexing (Ca^2+^/Mg^2+^). Serum-centric; longer assay time; limited whole-blood handling.
Johnson et al. 2025 [46]	Saliva	6	LIGMIS Electrochemical 3 min	0.004–3 mM LOD: 0.005 mM	Fast electrochemical sensing; portable integration. Electrode drift/calibration and matrix dependence may complicate routine use.
Li et al. 2025 [47]	Sweat	-	ISM Potentiometric Real-time	- LOD: 10 μM	Real-time wearable monitoring. Not designed for blood/serum; requires long-term stability management.
Ostad et al. 2017 [48]	Water	0	Xylidyl Blue Colorimetric 10 min	1–10 mg/dL LOD: 0.83 mg/dL	Simple paper device for low-complexity samples. Not representative of biofluid matrices; limited translation to whole blood/urine.

CPC: Cresolphthalein complexone; MTB: Methylthymol blue; OCP: o-Cresolphthalein complexone; LIGMIS: Laser-Induced Graphene Microfluidic Integrated Sensor.

## Data Availability

The data presented in this study are available on request from the corresponding author. The data are not publicly available due to patient privacy and ethical restrictions.
